# Correlations of erythrocytic oligomer α-synuclein levels with age, sex and clinical variables in patients with Parkinson’s disease

**DOI:** 10.3389/fnagi.2024.1437622

**Published:** 2024-07-31

**Authors:** Zhe Lu, Xiaohan Yu, Pengjie Li, Yiming Wang, Yeyun Deng, Xin Li, Chaodong Wang, Shun Yu

**Affiliations:** ^1^Department of Neurobiology and National Clinical Research Center for Geriatric Diseases, Xuanwu Hospital of Capital Medical University, Beijing, China; ^2^Department of Neurology, Xuanwu Hospital of Capital Medical University, Beijing, China

**Keywords:** oligomer, **α**-synuclein, erythrocyte, Parkinson’s disease, age, sex

## Abstract

**Introduction:**

Oligomeric alpha-synuclein in red blood cells (RBC-o-α-Syn) has been shown to be increased in patients with Parkinson’s disease (PD). However, factors that affect RBC-o-α-Syn levels remain to be elucidated. The aim of this study is to analyze the correlations between RBC-o-α-Syn levels and the age, sex and different clinical variables of patients with PD.

**Methods:**

167 patients with PD and 119 healthy controls (HC) were enrolled in this study. The patients with PD were diagnosed based on the MDS clinical diagnostic criteria for PD. All participants were evaluated for their clinical characteristics. Western blot analysis was used to examine the molecular sizes of RBC-o-α-Syn. A newly established chemiluminescent immunoassay was used to measure RBC-o-α-Syn levels.

**Results:**

Higher RBC-o-α-Syn levels were detected in PD patients than in HC subjects. The receiver operating characteristic (ROC) curve indicated that a cut off value of 55.29 ng/mg discriminated well between PD patients and HC subjects, with a sensitivity of 67.66% (95% CI: 60.24–74.29%), a specificity of 88.24% (95% CI: 81.22–92.86%), and an area under the curve (AUC) of 0.857. The levels of RBC-o-α-Syn were higher in female than male patients (*p* = 0.033). For different subtypes, the levels of RBC-o-α-Syn were higher in the MIX subtype than the tremor-dominant (TD) PD. In addition, the levels of RBC-o-α-Syn were higher in patients with than without cognitive impairment (*p* = 0.016), and negatively correlated with Mini-Mental State Examination (MMSE) scores (*r* = −0.156, *p* = 0.044).

**Conclusion:**

Our study demonstrates that RBC-o-α-Syn levels in patients with PD are higher than those in HC subjects and affected by the sex and the severity of cognitive impairment.

## 1 Introduction

Parkinson’s disease (PD) is a neurodegenerative disease characterized by movement disorders, which present tremor-dominant (TD), postural instability and gait difficulty (PIGD), and mixed (MIX) phenotypes (Stebbins et al., 2013). In addition to motor symptoms, PD patients also manifest various non-motor symptoms such as cognitive impairment (CI), sleep disorders, dysosmia, constipation, depression and anxiety (Jankovic, 2008; Schapira et al., 2017). So far, the diagnosis of PD is still based on the typical motor symptoms. Because the neuropathology of PD occurs much early than the motor symptoms, which are also overlapped with other parkinsonian syndromes, there is an urgent need for a diagnostic biomarker to assist PD clinical diagnosis.

The formation of Lewy bodies (LBs) with insoluble alpha-synuclein (α-Syn) fibers as the core within neurons is one of the main pathological features of Parkinson’s disease (Burre et al., 2015). Abnormal α-Syn aggregation is thought to play an important role in the onset and progression of PD. Although the α-Syn in the LBs exists in the form of insoluble fibers, a body of evidence supports that the oligomeric forms of α-Syn (o-α-Syn) is the most neurotoxic (Caughey and Lansbury, 2003; Bengoa-Vergniory et al., 2017). Therefore, detection of o-α-Syn is thought to better reflect the α-Syn pathology in PD patients.

Various methods have been established to detect o-α-Syn in cerebrospinal fluids (CSF) and blood plasma (Eusebi et al., 2016; Fayyad et al., 2019; Parnetti et al., 2019). These studies consistently demonstrate a significant increase in o-α-Syn levels in the CSF and plasma in PD patients. However, the concentrations of o-α-Syn detected vary greatly among different studies. One of the major reasons for the inconsistency is probably due to the contamination derived from hemolysis. Red blood cells (RBCs) contain abundant α-Syn that is 1,000 times higher than that in plasma (Barbour et al., 2008). A small amount of hemolysis can cause a significant increase of α-Syn in plasma and CSF (Hong et al., 2010; Youssef et al., 2021). Thus, direct detection of RBC-o-α-Syn is an option that not only can avoid hemolysis-derived contamination but also improve detection stability owing to the high α-Syn concentration in RBCs. In addition, evidence from previous studies suggests that the brain α-Syn can not only enter plasma through the blood–brain barrier but also be further transported into RBCs through a receptor-dependent endocytosis (Sui et al., 2014; Li et al., 2022), further supporting the feasibility of detecting RBC-o-α-Syn as a diagnostic biomarker for PD.

Several studies have been conducted to measure the levels of RBC-o-α-Syn (Wang et al., 2015; Daniele et al., 2018; Tian et al., 2019; Liu et al., 2022; Yu et al., 2022, 2023), which produced significantly different levels of RBC-o-α-Syn, although they were higher in PD patients than healthy controls (HC). In addition, the associations of RBC-o-α-Syn levels with the sex, age, and clinical variables remains elusive. One of the reasons may be due to the use of different standards in these studies, which were either the unpurified o-α-Syn or filamented α-Syn. Therefore, it is necessary to prepare a pure o-α-Syn standard with molecular sizes similar to those in RBCs for accurate measurement of RBC-o-α-Syn concentration.

In the present study, we established a chemiluminescent immunoassay (CLIA) for detecting RBC-o-α-Syn by using a stable purified o-α-Syn standard with molecular sizes similar to those in RBCs. With this new CLIA assay, we measured RBC-o-α-Syn levels in large cohorts of PD patients and HC subjects (HC) and analyzed the correlations of RBC-o-α-Syn with the sex, age, and various clinical variables.

## 2 Materials and methods

### 2.1 Participants

PD patients were recruited from Xuanwu Hospital of Capital Medical University. At the same time, HC participants were recruited from Community Health Service Center of Qinglonghu Town, Fangshan District, Beijing. Both PD patients and HC participants underwent evaluation by investigators, and re-evaluated by senior movement disorder specialists. All PD patients were diagnosed based on the MDS clinical diagnostic criteria for PD (Postuma et al., 2015). PD patients with the following conditions were excluded: (i) Parkinsonian syndromes caused by hypoxia, infectious, traumatic, cerebrovascular, metabolic or systemic diseases affecting the central nervous system; (ii) Parkinson’s plus syndromes, including dementia with Lewy bodies (DLB), multiple system atrophy (MSA), progressive supranuclear palsy (PSP), and corticobasal degeneration (CBD); (iii) ambiguous diagnosis due to unclear clinical or imaging features; (iv) a first degree relative with PD patients. HC participants with the following conditions were excluded: (i) motor symptoms such as tremor, bradykinesia, restless legs; (ii) history and symptoms of dysosmia and rapid eye movement behavioral disorder (RBD), or more than two other non-motor symptoms, including CI, constipation, depression, anxiety, according to the cut-off values of each scale for such symptoms; (iii) history and symptoms of stroke, dementia, hypoxia-related disorders and blood diseases. PD and HC subjects were matched for age and sex. Participants with incomplete or unclear demographic and clinical information were excluded from the study.

This study was approved by the Institutional Review Board and Ethics Committees of the participating hospitals. Before being included in the study, all participants or their legal guardians signed written informed consent.

### 2.2 Clinical evaluation

All participants were comprehensively assessed for demographic information and clinical characteristics by investigators. H&Y stage was used to assess the pathological progression of PD. PD patients were divided into different motor subtypes, including TD, PIGD and MIX, based on Movement Disorder Society sponsored revision of the Unified Parkinson’s Disease Rating Scale Part III (MDS-UPDRS III) scores, specifically, the ratio of total tremor score/total PIGD score in MDS-UPDRS III, whose value ≥1.15 classified as TD, ≤ 0.9 classified as PIGD and 0.9–1.15 classified as MIX. Non-motor subtypes were defined by the existence of CI, RBD, dysosmia, constipation, depression and anxiety, according to cut off points on scores in scales quantifying these symptoms: a cut-off value of 26 on the Mini-mental State Examination (MMSE) for CI, a cut-off value of 19 on the Rapid Eye Movement Sleep Behavior Disorder Questionnaire-Hong Kong (RBDQ-HK) for RBD, a cut-off value of 22 on the Argentine Hyposmia Rating Scale (AHRS) for dysosmia, two terms on the Diagnostic Criteria (ROME III) for constipation, a cut-off value of 8 on the Hamilton Depression Scale (HAMD) for depression, and a cut-off value of 7 on the Hamilton Anxiety Scale (HAMA) for anxiety.

### 2.3 RBC samples

When participants underwent evaluation, whole blood samples (10 mL) were collected into EDTA anti-coagulant tubes. The blood was centrifuged at 4°C and 1,500 × *g* for 15 min. The upper and middle layers, containing plasma and white blood cells, were removed. And the lower layer, containing RBCs, was washed with 10 mM PBS (1.9 mM NaH_2_PO_4_, 8.1 mM Na_2_HPO_4_, 153.85 mM NaCl, pH 7.4) for three times. Finally, the isolated RBC samples were collected and preserved in a freezer (−80°C). 48 h later, RBC samples were taken out and thawed on ice, and stored at −80°C again. Protein concentrations were measured with a bicinchoninic acid (BCA) protein assay kit (23,225, Thermo Fisher, United States) before the assay.

### 2.4 Preparation of o-α-Syn standard

Recombinant human α-Syn and o-α-Syn were prepared according to the methods described already (Li et al., 2020). In brief, pET-15b-NACP plasmids were transformed into BL21 (DE3) and the expressed α-Syn proteins were purified with high performance liquid chromatography. To prepare o-α-Syn, purified human α-Syn (4 mg/mL) was dissolved in 0.01 M PBS and incubated by constant shaking at 37°C (1,000 rpm) for 4 days on an Eppendorf Thermomixer Comfort (AG2233, Eppendorf, Germany). O-α-Syn (34–170 kDa) in the mixture was separated with sodium dodecyl sulfate-polyacrylamide gel electrophoresis (SDS-PAGE) and recovered from the gel with electrodialysis. Purified o-α-Syn was stored at −20°C.

### 2.5 Western blot

Total protein concentrations of RBCs and purified o-α-Syn were measured using the BCA Protein Assay Kit. RBCs were adjusted to 10 mg/mL total protein concentrations with 0.01 M PBS, while purified o-α-Syn were adjusted to 100 μg/mL. RBCs (200 μg/sample) and purified o-α-Syn (5 μg/sample) were separated with SDS-PAGE and transferred onto polyvinylidene fluoride (PVDF) membranes (IPVH00010, Millipore, United States). The PVDF membranes were blocked with 5% non-fat milk dissolved in triethanolamine buffered saline containing 0.1% Tween-20 (TBST) for 1 h. The membranes were probed with mouse monoclonal anti-human-α-Syn antibody (3D5, 1:20000) at 25°C for 16 h. After washed with TBST, the membranes were probed with horseradish peroxidase (HRP)-conjugated goat anti-mouse IgG (ZB-5305, ZSGB-BIO, China, 1:10000) at 25°C for 1 h. The blots were visualized by enhanced chemiluminescence (ECL) kit (20,063, Millipore, USA). β-actin in RBCs were detected as internal reference.

### 2.6 Chemiluminescent immunoassay

To detect o-α-Syn, alkaline phosphatase (AP)-labeled and unlabeled mouse monoclonal anti-α-Syn (3D5) antibodies were used as the detection and capture antibodies, respectively. The calibration curves were constructed using a series of concentrations of o-α-Syn. A CLIA plate was coated with 100 μL/well primary anti-α-Syn antibody (1 μg/mL) in coating buffer (0.2 M NaHCO_3_, pH 9.6) and incubated at 4°C for 16 h. After washing with PBS containing 0.05% Tween-20 (PBST) and blocked with 1% BSA in PBST at 37°C for 2 h, 100 μL/well RBC samples (1:100 diluted) or different concentrations of o-α-Syn standards were added and incubated at 37°C for 1 h. Then the wells were washed with PBST, 100 μL of the AP anti-α-Syn 3D5 (1 μg/mL) in 1% BSA was added to the wells, and incubated at 37°C for 1 h. After washed with PBST, 100 μL/well of AP-catalyzed chemiluminescence liquid (APCL-1, APIS, Beijing, China) was added to each well. Then, chemiluminescent intensity was measured using fluorescence analyzer (Flx800, Biotek, United States). All the samples were tested in triplicates within the same assay and on the same day.

### 2.7 Statistical analysis

Demographic, clinical characteristics, and o-α-syn data were divided into different types. The Chi-squared test was performed to compare the distribution of categorical variables among groups. Measurement data were expressed as means ± standard deviation (SD). For normally distributed data, Student’s *t*-test was used for comparisons between two groups. And for skewed data, Kruskal-Wallis test was used for comparisons among groups. Level data were expressed as Median (lower quartile, upper quartile), and for which, Kruskal-Wallis test was used for comparisons between two groups.

Receiver operating characteristic (ROC) curves were constructed and the area under the curve (AUC) was calculated to evaluate the sensitivity and specificity of o-α-syn levels in RBCs as diagnostic biomarker for PD, identifying the best cut-off value as the point with the highest Youden index. Correlations of clinical variables such as MDS-UPDRS III scores and MoCA (Montreal Cognitive Assessment) scores with RBC-o-α-Syn concentrations were examined by linear regression models and Pearson correlation analysis.

All statistical analyses were performed with GraphPad Prism 8 (GraphPad, San Diego, CA, United States). *p*-values <0.05 were considered as statistically significant.

## 3 Results

The demographic and clinical data for the participants in this study are shown in Table 1, which included 119 HC subjects and 167 PD patients recruited from July 2021 to July 2023. All participants had complete demographic data with the male/female ratios of 52/67 in HC group and 80/87 in PD group. The average age of education in PD group was higher than that in HC group. All participants were evaluated for their non-motor symptoms, which showed lower scores of MMSE and MoCA and higher scores of RBDQ-HK, AHRS, HAMD, and HAMA in PD than in HC groups. The PD patients was also evaluated for their age at onset (59.3 ± 9.4), disease duration (4.6 ± 3.9), H&Y stage [2 (1,2)], and MDS-UPDRS III scores (26.5 ± 14.5).

**Table 1:** Demographic and clinical data for participants.

Variables	HC	PD
Sex (male/female)	52/67	80/87
Age (y)	66.4 ± 5.6	63.8 ± 8.7
Education (y)	8.9 ± 5.1	9.6 ± 4.4
Age at onset (y)	NA	59.3 ± 9.4
Disease duration (y)	NA	4.6 ± 3.9
H&Y stage	NA	2 (1, 2)
MDS-UPDRS III scores	NA	26.5 ± 14.5
MMSE	26.7 ± 2.5	24.1 ± 5.4
MoCA scores	22.8 ± 4.0	19.7 ± 6.2
RBDQ-HK scores	5.9 ± 5.0	16.1 ± 14.6
AHRS scores	24.0 ± 0.1	19.5 ± 7.0
HAMD scores	1.4 ± 2.2	6.2 ± 6.2
HAMA scores	3.7 ± 3.7	8.1 ± 7.3

Measurement data were expressed as means ± SD, level data were expressed as Median (lower quartile, upper quartile). HC, Healthy control; PD, Parkinson’s disease; MDS-UPDRS III, Movement Disorder Society-sponsored revision of the Unified Parkinson’s Disease Rating Scale Part III; MMSE, Mini-Mental State Examination; MoCA, Montreal Cognitive Assessment; RBDQ-HK, Rapid eye movement sleep behavior disorder questionnaire-Hong Kong; AHRS, Argentine Hyposmia Rating Scale; HAMD, Hamilton Depression Scale; HAMA, Hamilton Anxiety Scale. NA, not applicable.

**Figure 1:**
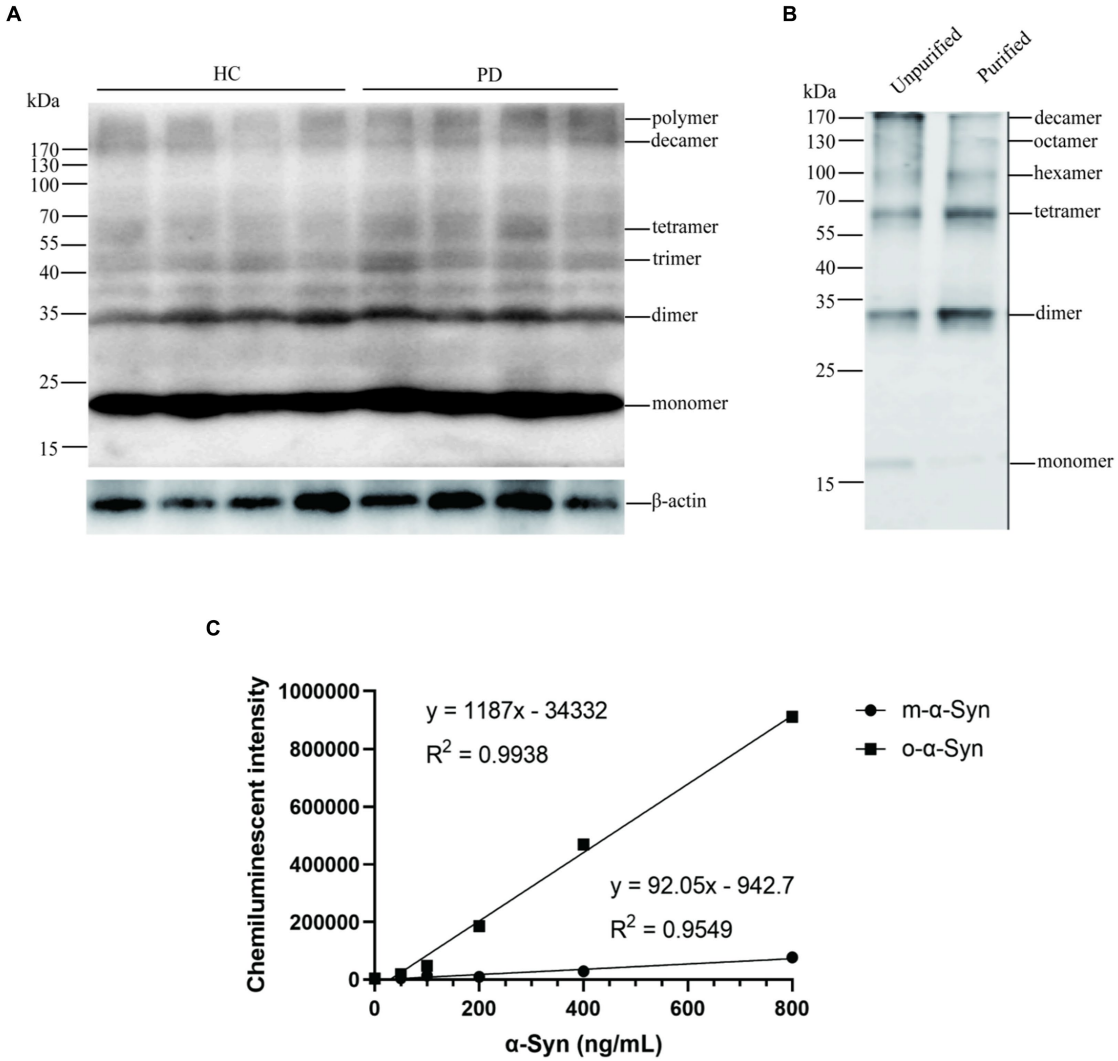
Identification of o-α-Syn standards. **(A)** Western blot revealed α-Syn monomers, dimers, trimers, tetramers, decamers, and polymers in (n = 4) and PD (n = 4) RBCs. **(B)** The mixture containing α-Syn oligomers similar to those in RBCs were prepared, purified and used as the standard. **(C)** Standard curve between concentrations of α-Syn oligomers as well as monomers and chemiluminescent intensity was ploted.

We first analyzed the molecular sizes of RBC-o-α-Syn with Western blot using the RBC lysates of PD patients and HC subjects. In addition to monomer α-Syn, we detected different sizes of oligomer in the RBC lysates, which included dimers (34 kDa), trimers (51 kDa), tetramers (68 kDa), decamers (170 kDa), and bigger polymers (Figure 1A).

Based on the above results, we prepared o-α-Syn standards with molecular sizes similar to those found in RBC lysates (Figure 1B). We used our newly established CLIA method to detect the o-α-Syn standards. We showed that the chemiluminescent intensity was linearly correlated with the o-α-Syn concentration (R^2^ = 0.9938) (Figure 1C).

**Figure 2:**
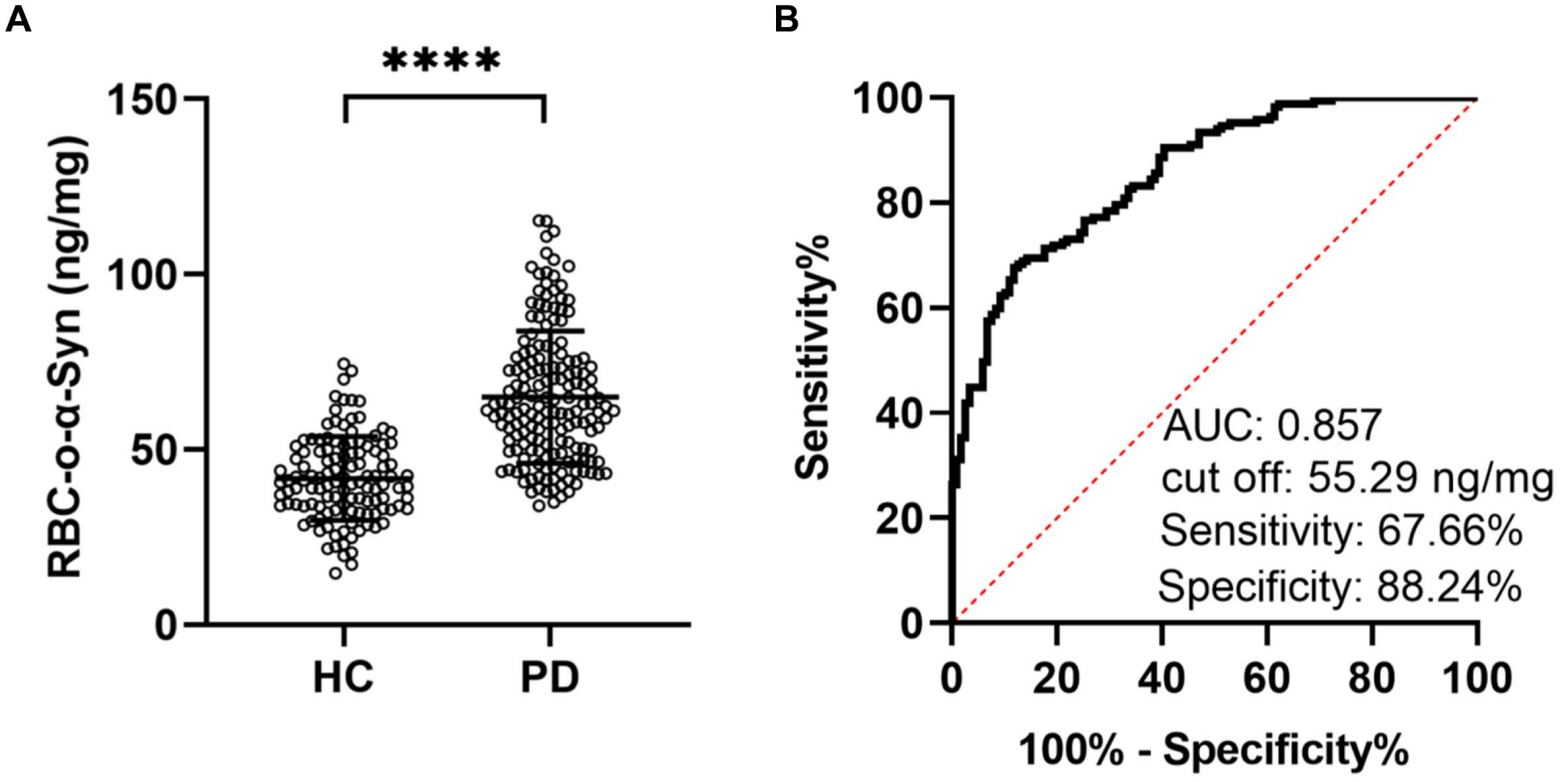
RBC-o-α-Syn levels measured in HC and PD groups. **(A)** RBC-o-α-Syn levels in PD (*n* = 167) vs. HC (*n* = 119). ^****^p < 0.0001. **(B)** ROC curve showing the sensitivity, specificity and area under the curve (AUC) for RBC-o-α-Syn in discriminating PD from HC.

We then used the CLIA method to measure the levels of RBC-o-α-Syn. We showed that the levels of RBC-o-α-Syn were significantly higher in PD patients than HC subjects (64.92 ± 18.81 ng/mg vs. 41.72 ± 11.87 ng/mg; *p <* 0.0001) (Figure 2A). ROC curve analysis showed that a cut off value of 55.29 ng/mg discriminated PD patients well from HC subjects, with a sensitivity of 67.66% (95% CI: 60.24–74.29%), a specificity of 88.24% (95% CI: 81.22–92.86%), and an AUC of 0.857 (Figure 2B).

**Figure 3:**
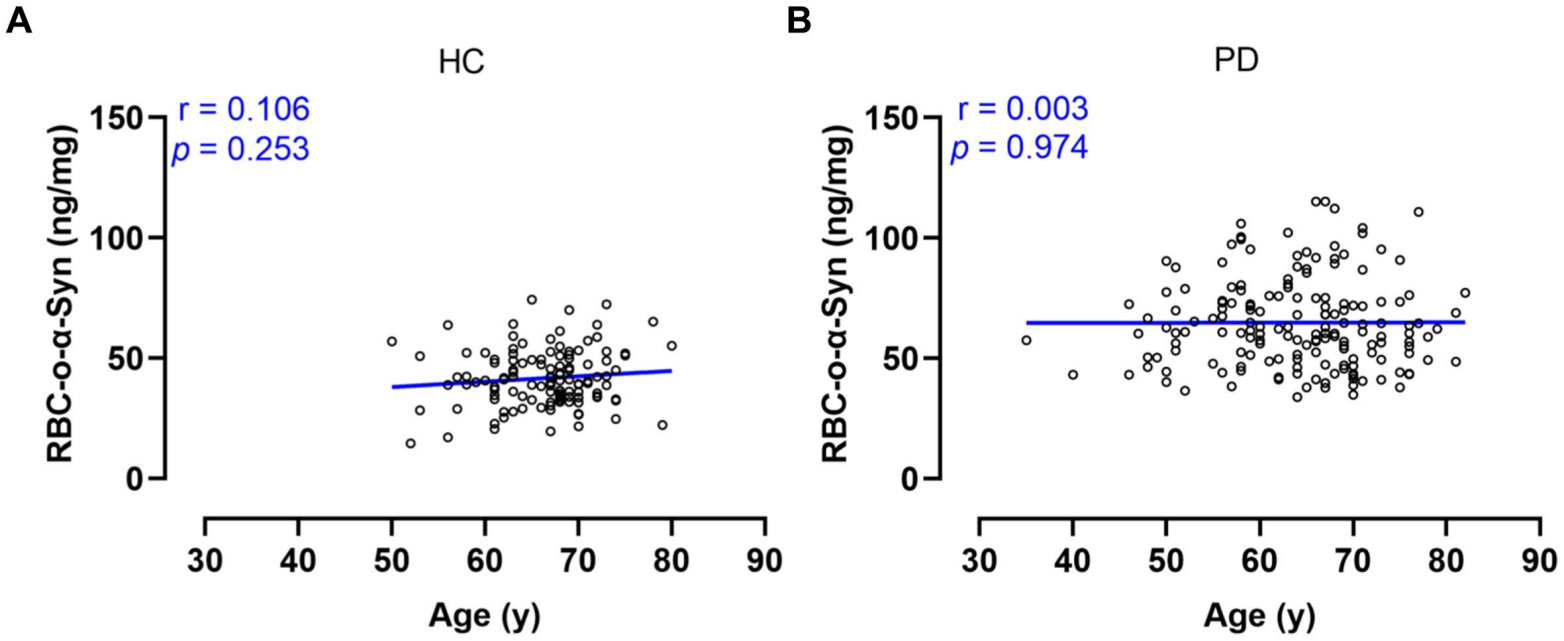
Correlations between RBC-o-α-Syn levels and ages **(A)** in HC **(B)** in PD.

**Figure 4:**
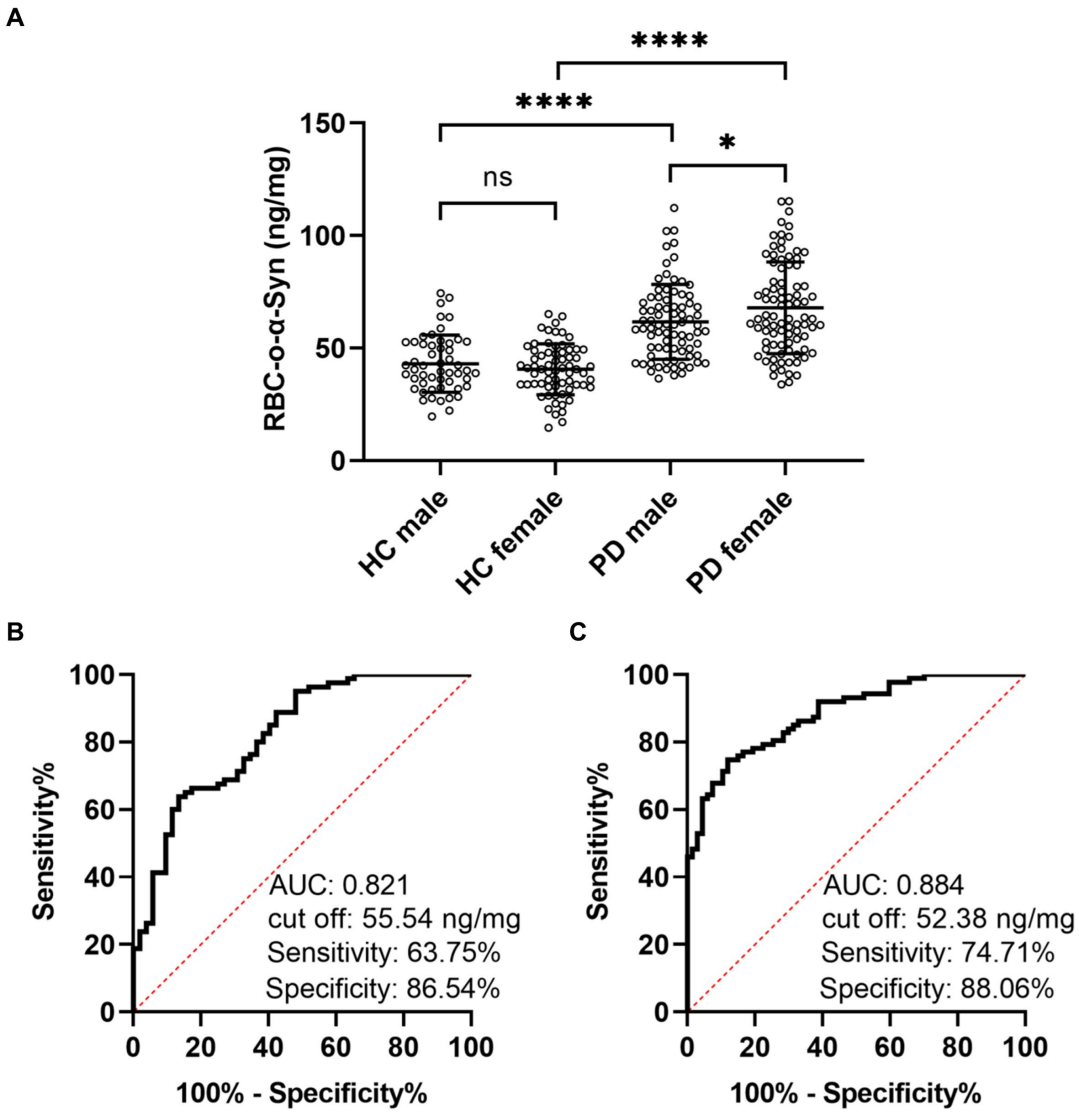
Difference of RBC-o-α-Syn levels in HC and PD participants with different sexes. **(A)** RBC-o-α-Syn levels in male (PD: 80, HC: 52) and female (PD: 87, HC: 67) participants. ns, no significance. ^*^p < 0.05. ^****^p < 0.0001. **(B,C)** ROC curve showing the sensitivity, specificity and area under the curve (AUC) for RBC-o-α-Syn in discriminating PD form HC in male and female participants.

Linear regression model analysis showed that the RBC-o-α-Syn levels in HC (r = 0.106, *p* = 0.253) and PD (r = 0.003, *p* = 0.974) groups were not correlated with ages (Figures 3A,B). As for sex differences, the RBC-o-α-Syn levels in the female participants in PD group were significantly higher than those in the male participants (*p* = 0.033). The difference was not detected in HC group (*p =* 0.263) (Figure 4A). In the male participants, RBC-o-α-Syn levels in PD and HC groups were 61.68 ± 16.48 ng/mg and 43.11 ± 12.58 ng/mg, respectively (*p <* 0.0001) (Figure 4A). The AUC of ROC curve was 0.821 under a cut off value of 55.54 ng/mg, with a sensitivity of 63.75% (95% CI: 52.81–73.43%) and a specificity of 86.54% (95% CI: 74.73–93.32%) in discriminating PD patients from HC subjects (Figure 4B). While in the female participants, RBC-o-α-Syn levels in PD and HC groups were 67.91 ± 20.27 ng/mg and 40.63 ± 11.18 ng/mg, respectively (*p <* 0.0001) (Figure 4A). The AUC of ROC curve was 0.884 under a cut off value of 52.38 ng/mg, with a sensitivity of 74.71% (95% CI: 64.67–82.67%) and a specificity of 88.06% (95% CI: 78.17–93.82%) in separating PD patients with HC subjects (Figure 4C).

**Figure 5:**
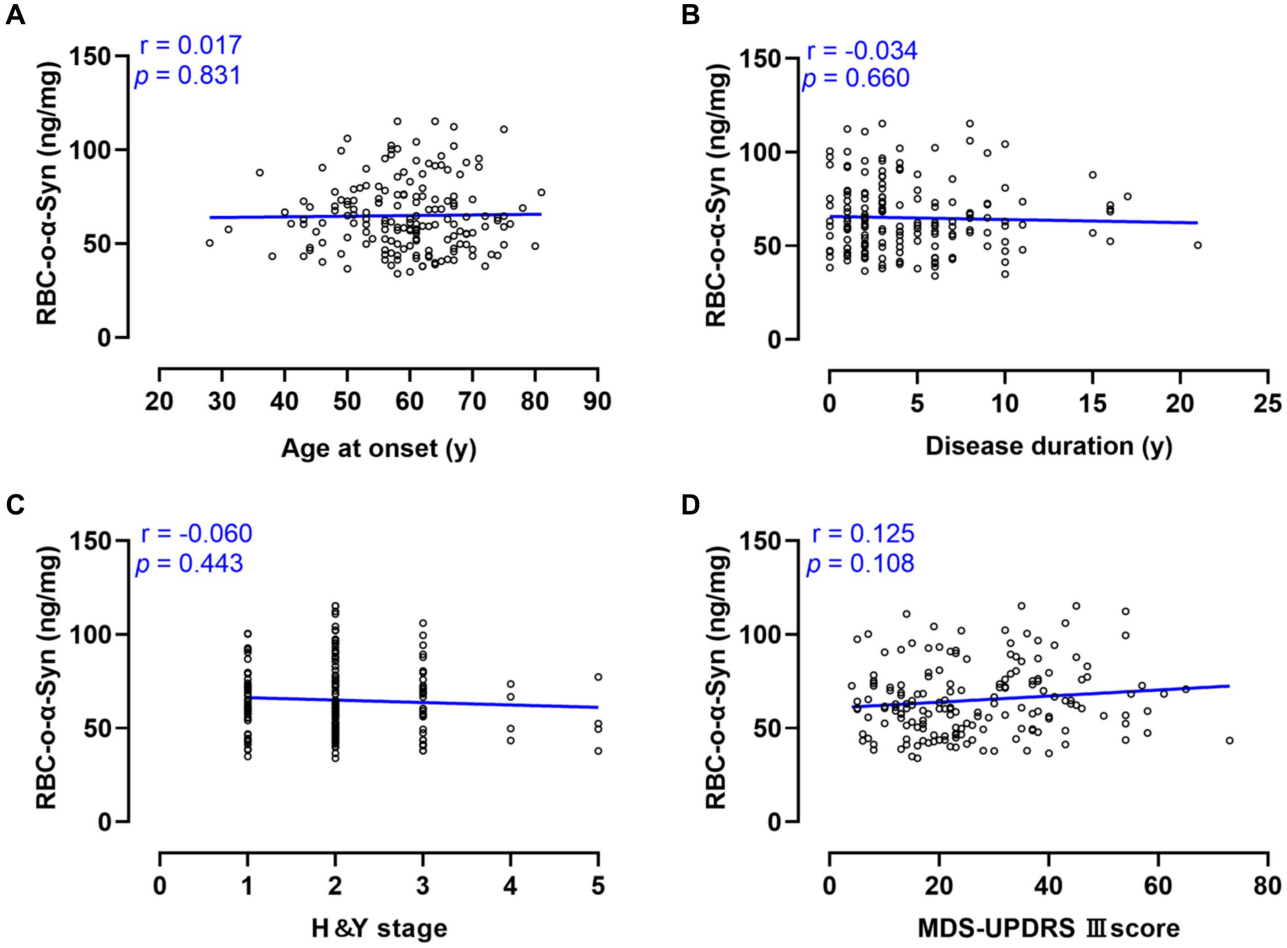
Correlations between RBC-o-α-Syn and PD progression and motor symptoms. **(A)** Age at onset. **(B)** Disease duration. **(C)** H&Y stage. **(D)** MDS-UPDRS III score.

Linear regression analysis showed that RBC-o-α-syn levels in PD patients were not correlated with ages at onset (*r* = 0.017, *p* = 0.831), disease duration (*r* = −0.034, *p* = 0.660), H-Y stages (*r* = −0.060, *p* = 0.443) and MDS-UPDRS III scores (*r* = 0.125, *p* = 0.108) (Figure 5).

For different subtypes, RBC-o-α-Syn levels in MIX subtype (71.77 ± 17.76 ng/mg) were higher than those in TD (63.24 ± 18.46 ng/mg, *p* = 0.036) (Table 2). Moreover, as an exploratory analysis, we compared the differences in RBC-o-α-Syn levels among different subtypes stratified by several variables. As a result, only in patients with age at onset <60 years or disease duration between 6 and 10 years, the RBC-o-α-Syn levels in the MIX subtype were higher than those in the PIGD subtype (Table 2).

**Table 2:** Overall and clinical variable-stratified comparisons of RBC-o-α-Syn levels among subtypes of PD.

Variables	All PD	TD	PIGD	MIX	*P* (TD vs. PIGD)	*P* (TD vs. MIX)	*P* (PIGD vs. MIX)
Demographic and clinical data							
Sex (male/female)	80/87	41/45	26/27	13/15	> 0.999	> 0.999	> 0.999
Age (y)	63.8 ± 8.7	63.4 ± 8.7	64.3 ± 8.8	64.3 ± 8.4	0.573	0.647	0.999
Age at onset (y)	59.3 ± 9.4	59.3 ± 9.2	59.4 ± 9.8	58.8 ± 9.5	0.970	0.796	0.796
Disease duration (y)	4.6 ± 3.9	4.1 ± 3.2	4.9 ± 4.3	5.5 ± 5.0	0.213	0.089	0.582
H&Y stage	2 (1, 2)	2 (1, 2)	2 (2, 3)	2 (2, 2)	**0.002**	0.077	0.437
RBC-o-α-Syn in patients (ng/mg)							
RBC-o-α-Syn (ng/mg)	64.92 ± 18.81	63.24 ± 18.46	64.03 ± 19.12	71.77 ± 17.76	0.810	**0.036**	0.083
RBC-o-α-Syn in patients with different sexes (ng/mg)							
Male	61.68 ± 16.48	59.58 ± 16.49	62.34 ± 17.20	66.97 ± 13.43	0.521	0.156	0.413
Female	67.91 ± 20.27	66.57 ± 19.50	65.67 ± 20.68	75.94 ± 19.87	0.854	0.120	0.135
RBC-o-α-Syn in patients stratified by age (ng/mg)							
< 65 (y)	65.30 ± 17.44	65.72 ± 18.53	61.40 ± 14.65	71.36 ± 16.66	0.327	0.337	0.073
≥ 65 (y)	64.56 ± 20.04	60.64 ± 18.02	66.38 ± 22.11	72.13 ± 18.65	0.244	**0.044**	0.408
RBC-o-α-Syn in patients stratified by age at onset (ng/mg)							
< 60 (y)	65.75 ± 18.15	66.62 ± 20.41	61.25 ± 13.88	72.46 ± 16.02	0.245	0.360	**0.033**
≥ 60 (y)	64.16 ± 19.37	60.31 ± 16.01	66.92 ± 23.00	71.18 ± 19.12	0.163	**0.037**	0.557
RBC-o-α-Syn in patients stratified by disease duration (ng/mg)							
0–2 (y)	64.88 ± 18.61	63.76 ± 17.24	66.85 ± 22.08	63.44 ± 10.41	0.577	0.962	0.687
3–5 (y)	67.47 ± 19.36	65.87 ± 18.87	60.72 ± 17.51	77.37 ± 18.41	0.482	0.097	0.067
6–10 (y)	62.39 ± 18.88	60.22 ± 19.36	60.08 ± 16.36	79.68 ± 20.81	0.982	0.062	0.055
> 10 (y)	65.07 ± 11.85	52.33 ± 4.52	71.71 ± 8.80	63.15 ± 11.79	0.054	0.364	0.308
RBC-o-α-Syn in patients stratified by H&Y stages (ng/mg)							
1	64.04 ± 16.62	65.70 ± 17.26	57.25 ± 16.42	68.36 ± 6.46	0.199	0.744	0.198
2	66.42 ± 20.11	63.95 ± 19.49	67.18 ± 21.30	71.73 ± 18.79	0.536	0.170	0.494
3	64.27 ± 18.47	57.08 ± 15.60	66.28 ± 16.36	80.62 ± 21.63	0.163	**0.040**	0.196
4–5	56.31 ± 13.49	43.35	56.23 ± 13.98	62.98 ± 10.50	-	-	0.624

TD, Tremor-dominant subtype; PIGD, postural instability and gait difficulty subtype. Bold: *p* < 0.05.

**Figure 6:**
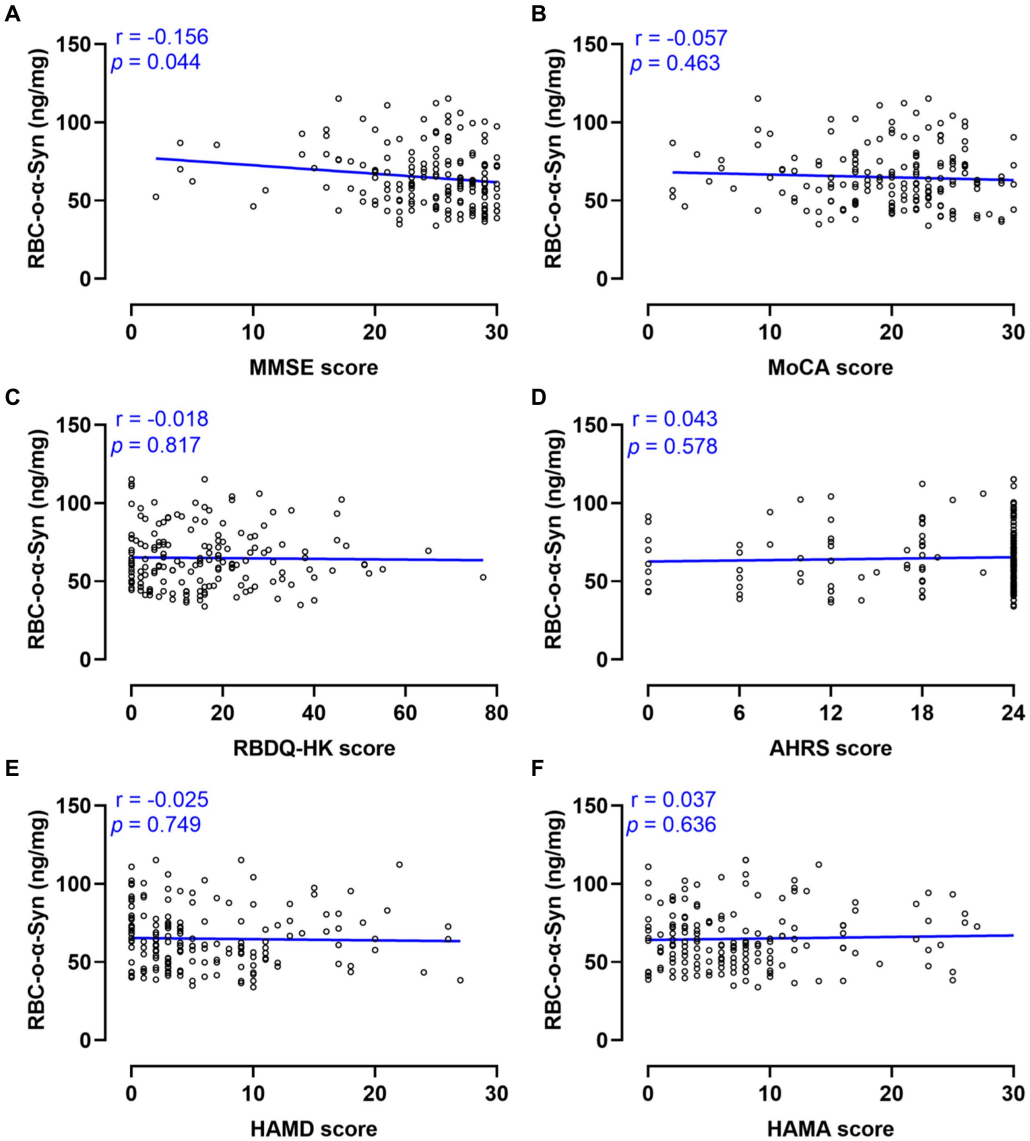
Correlations between RBC-o-α-Syn and non-motor symptoms. **(A)** MMSE scores. **(B)** MoCA scores. **(C)** RBDQ-HK scores. **(D)** AHRS scores. **(E)** HAMD scores. **(F)** HAMA scores.

Linear regression analysis showed that the RBC-o-α-Syn levels in PD patients were weak negatively correlated with MMSE scores (*r* = −0.156, *p* = 0.044), but not with MoCA, RBDQ-HK, AHRS, HAMA scores (*p* > 0.05) (Figure 6). As for non-motor subtypes, there was a significant difference in the levels of RBC-o-α-Syn between patients with cognitive impairment (CI) and those without it (Supplementary Table S1). And the RBC-o-α-Syn levels did not differ between patients with or without RBD, dysosmia, constipation, depression, and anxiety (Supplementary Tables S2–S5).

## 4 Discussion

In the present study, we established a new immunoassay to measure RBC-o-α-Syn levels. Similar to our previously established ELISA, we used the same anti-α-Syn monoclonal antibody as the capture and detection antibodies. However, in the present assay, the detection antibody was conjugated with AP, which catalyzed the chemiluminescence substrate (APCL-1) to emit fluorescence. This modification greatly shortened the assay time with equivalent sensitivity of the previously established ELISA method (Li et al., 2022). The assay time of CLIA was 3 h compared with 8 h of ELISA. In addition, we used the purified α-Syn oligomer mixtures with molecular sizes similar to those in RBCs as the standard. Compared with some previous studies that used α-Syn filament as the standard (Tian et al., 2019; Yu et al., 2022, 2023), the present measurement may better reflect the true concentration of RBC-o-α-Syn.

Our present results showed that RBC-o-α-Syn levels were significantly increased in PD patients compared with HC subjects and could discriminate well between PD patients and HC subjects, with a sensitivity of 67.66%, a specificity of 88.24%, and an AUC of 0.857. These results are consistent with previous findings (Wang et al., 2015; Tian et al., 2019; Yu et al., 2022), suggesting a good correlation of RBC-o-α-Syn with PD pathology. It is worth noting that the concentrations of RBC-o-α-Syn we measured were 64.92 ± 18.81 ng/mg in PD and 41.72 ± 11.87 ng/mg in HC, which were in contrast to the concentrations of 29.0 ± 19.8 ng/mg in PD and 15.4 ± 7.4 ng/mg in HC measured by ELISA as well as 218.1 ± 86.5 ng/mL in PD and 135.0 ± 42.2 ng/mL in HC measured by electrochemiluminescence assay (Wang et al., 2015; Yu et al., 2022). This may be due to the different sensitivity of the methods and the different standards used. For example, some studies used unpurified o-α-Syn or α-Syn filaments as the standard, which was not identical in molecular sizes to the o-α-Syn in RBCs (Tian et al., 2019; Yu et al., 2022, 2023).

Changes in o-α-Syn levels have been investigated in the other bodily fluids of PD patients, such as CSF, plasma, serum and saliva. For example, studies on CSF showed a significant increase in o-α-Syn levels in the CSF of PD patients compared with those in HC (Ganguly et al., 2021), with a sensitivity of 89%, a specificity of 48%, and an AUC of 0.72. Using the CSF o-α-Syn/total α-Syn ratio slightly increased the sensitivity (82%), specificity (64%), and AUC of 0.78% (Parnetti et al., 2014). Levels of o-α-Syn levels in plasma and serum have been also investigated. Although some studies showed an increase in the levels of o-α-Syn in the plasma and serum of PD patients, a meta-analysis shown that plasma and serum o-α-Syn levels were not statistically different between PD and HC groups. This is attributed to the small quantity of studies and the large heterogeneity (Zubelzu et al., 2022). The contamination derived from hemolysis may be one of the reasons for the large heterogeneity since RBCs contain 1,000 times α-Syn plasma (Barbour et al., 2008; Hong et al., 2010; Youssef et al., 2021). In addition, salivary o-α-Syn was also shown to be increased (Vivacqua et al., 2016). Because of only a few studies, its sensitivity, specificity and AUC in discriminating PD and HC remain to be determined. Taken together, the change of RBC-o-α-Syn measured in the present study are identical to the changes measured in other body fluids, with similar or even better diagnostic performance. However, detection of RBC-o-α-Syn could be more stable and reproducible due to the high concentration of RBC-o-α-Syn and the avoidance of hemolysis-derived contamination.

Because the prevalence of PD is related to sex and age, we further analyzed if RBC-o-α-Syn levels were affected by sex and age. We showed that RBC-o-α-Syn levels in female PD patients were significantly higher than those in male PD patients, and this sex difference was not detected in HC subjects. These results support the report that the female PD patients have faster disease progression and significantly shorter time from onset to severe disability compared to the male PD patients (Dahodwala et al., 2018), but contradictory to the epidemiological study results that PD affects men twice more often than women (Wooten et al., 2004; Picillo and Fasano, 2015; Cerri et al., 2019; Vaidya et al., 2021). This contradiction indicates that the factors promoting PD onset are complex and o-α-Syn aggregation is only one of the factors. Since aging is the most important risk factor for PD (Nussbaum and Ellis, 2003; de Lau and Breteler, 2006), we also analyzed the correlation between age and RBC-o-α-Syn. However, there were no correlations between RBC-o-α-Syn levels and ages in both HC and PD participants. The results are inconsistent with the observations in both mouse and non-human primate models showing that the brain o-α-Syn levels and age are positively correlated (Chen et al., 2016; Ho et al., 2020). This may be due to the lack of sufficient young participants in this study. To explore the correlation between RBC-o-α-Syn levels and ages, further study on participants in wide and strictly controlled age groups is needed.

We further analyzed the correlations between RBC-o-α-Syn levels and different clinical variables. Significant correlations of RBC-o-α-Syn levels were not detected with the ages at onset, disease duration, H&Y stages and MDS-UPDRS III scores, which is consistent with results reported in previous studies (Wang et al., 2015). These results may indicate that the change of aggregation degree of α-synuclein in PD progression is a complex process, rather than a simple linear increase or decrease.

We next compared the levels of RBC-o-α-Syn in different PD subtypes. We found that the levels of RBC-o-α-Syn were significantly higher in the MIX subtype patients than those in the TD subtype. In addition, in patients with ages <60 years or disease duration from 6 to 10 years, the RBC-o-α-Syn levels in the MIX subtype were higher than those in the PIGD subtype.

Cognitive impairment is common in PD, whose incidence rate increases with age, disease duration, and pathological progress (Litvan et al., 2012). Long term follow-up studies have shown that up to 80% of PD patients ultimately exhibit symptoms of dementia (Aarsland et al., 1996; Hely et al., 2008). Our study demonstrated that RBC-o-α-Syn levels in PD patients with Cognitive impairment were higher than those without it. In addition, we found that RBC-o-α-Syn levels in PD patients were weak negatively correlated with MMSE scores, but not with MoCA scores. It is found that MoCA exhibits better performance in the differential diagnosis of healthy controls and PD dementia, but the MMSE score presents higher correlation with the severity of PD cognitive impairment (Lessig et al., 2012). This may explain the correlation of RBC-o-α-Syn with MMSE and not MoCA.

There are some limitations in this study. One is the absence of patients with other synucleinopathies and parkinsonian syndromes such as multiple system atrophy (MSA), dementia with Lewy bodies, progressive supranuclear paralysis, corticobasal degeneration. Second is the lack of apparent correlations between RBC-o-α-Syn levels with PD progression of PD. Longitudinal cohort study from prodromal to clinical stage is needed to further determine the correlations. Third, correlations between RBC-o-α-Syn levels and age, subtype, H&Y stage need to be verified in further studies with control for multiple testing.

In conclusion, our study demonstrates that RBC-o-α-Syn levels in patients with PD patients were higher than those in HC subjects and can be a potential diagnostic biomarker for PD. RBC-o-α-Syn levels were higher in female than in male PD patients. In addition, RBC-o-α-Syn levels are positively correlated with the severity of cognitive impairment.

## Data availability statement

The original contributions presented in the study are included in the article/Supplementary material, further inquiries can be directed to the corresponding author.

## Ethics statement

The studies involving humans were approved by the Institutional Review Board and Ethics Committees of XuanWu hospital. The studies were conducted in accordance with the local legislation and institutional requirements. Written informed consent for participation in this study was provided by the participants' legal guardians/next of kin.

## Author contributions

ZL: Conceptualization, Data curation, Formal analysis, Methodology, Validation, Writing – original draft, Writing – review & editing. XY: Conceptualization, Data curation, Formal analysis, Methodology, Validation, Writing – review & editing. PL: Data curation, Formal analysis, Methodology, Validation, Writing – review & editing. YW: Methodology, Resources, Validation, Writing – review & editing. YD: Methodology, Resources, Validation, Writing – review & editing. XL: Methodology, Resources, Validation, Writing – review & editing. CW: Conceptualization, Funding acquisition, Investigation, Project administration, Resources, Supervision, Validation, Writing – review & editing. SY: Conceptualization, Data curation, Formal analysis, Funding acquisition, Investigation, Methodology, Project administration, Resources, Supervision, Validation, Writing – original draft, Writing – review & editing.
