# Supplementary material for: Correlations of erythrocytic oligomer α-synuclein levels with age, sex and clinical variables in patients with Parkinson’s disease
Source: Front Aging Neurosci. 2024 Jul 31;16:1437622. doi: 10.3389/fnagi.2024.1437622 (PMC11322579; doi:10.3389/fnagi.2024.1437622)
Supplement: Supplementary file 1 [file Table_1.DOCX]

**Supplementary Table 1 |** Overall and clinical variable-stratified comparisons of RBC-o-α-Syn levels in PD patients with or without CI.

| Variables | CI (-) | CI (+) | *P-value* |
| --- | --- | --- | --- |
| **Demographic and clinical data** | | | |
| Sex (male/female) | 35/32 | 45/55 | 0.430 |
| Age (y) | 61.2 ± 9.2 | 65.6 ± 7.8 | **0.001** |
| Age at onset (y) | 56.8 ± 9.7 | 60.9 ± 8.8 | **0.005** |
| Disease duration (y) | 4.4 ± 3.7 | 4.7 ± 4.1 | 0.641 |
| H＆Y stage | 2 (1, 2) | 2 (2, 3) | **0.026** |
| **RBC-o-α-Syn in patients (ng/mg)** | | | |
| RBC-o-α-Syn (ng/mg) | 60.64 ± 17.03 | 67.80 ± 19.39 | **0.016** |
| **RBC-o-α-Syn in patients with different sexes (ng/mg)** | | | |
| Male | 56.96 ± 14.59 | 65.35 ± 16.93 | **0.024** |
| Female | 64.66 ± 18.54 | 69.80 ± 20.99 | 0.259 |
| **RBC-o-α-Syn in patients stratified by age (ng/mg)** | | | |
| < 65 (y) | 63.52 ± 17.88 | 66.92 ± 16.88 | 0.384 |
| ≥ 65 (y) | 56.62 ± 14.88 | 68.46 ± 21.07 | **0.010** |
| **RBC-o-α-Syn in patients stratified by age at onset (ng/mg)** | | | |
| < 60 (y) | 63.15 ± 17.72 | 68.49 ± 18.21 | 0.194 |
| ≥ 60 (y) | 56.67 ± 15.07 | 67.35 ± 20.10 | **0.018** |
| **RBC-o-α-Syn in patients stratified by disease duration (ng/mg)** | | | |
| 0-2 (y) | 61.12 ± 17.06 | 67.78 ± 19.25 | 0.165 |
| 3-5 (y) | 57.42 ± 16.08 | 72.20 ± 18.96 | **0.011** |
| 6-10 (y) | 61.15 ± 17.30 | 63.51 ± 21.38 | 0.698 |
| > 10 (y) | 79.77 ± 8.08 | 61.80 ± 9.92 | 0.059 |
| **RBC-o-α-Syn in patients stratified by H＆Y stages (ng/mg)** | | | |
| 1 | 60.64 ± 15.86 | 67.96 ± 16.61 | 0.157 |
| 2 | 59.49 ± 15.05 | 70.20 ± 21.47 | **0.019** |
| 3 | 63.09 ± 21.94 | 65.24 ± 14.96 | 0.757 |
| 4-5 | - | 56.31 ± 13.49 | - |

*CI, cognitive impairment; CI (-): MMSE scores ≥ 26; CI (+): MMSE scores < 26. Bold, p < 0.05. -, not available.*

**Supplementary Table 2 |** Overall and clinical variable-stratified comparisons of RBC-o-α-Syn levels in PD patients with or without RBD.

| Variables | RBD (-) | RBD (+) | *P-value* |
| --- | --- | --- | --- |
| **Demographic and clinical data** | | | |
| Sex (male/female) | 47/58 | 33/29 | 0.337 |
| Age (y) | 63.3 ± 8.7 | 64.8 ± 8.5 | 0.270 |
| Age at onset (y) | 59.3 ± 9.6 | 59.2 ± 9.1 | 0.997 |
| Disease duration (y) | 4.0 ± 3.4 | 8.8 ± 4.3 | **< 0.0001** |
| H＆Y stage | 2 (1, 2) | 2 (2, 3) | **0.026** |
| **RBC-o-α-Syn in patients (ng/mg)** | | | |
| RBC-o-α-Syn (ng/mg) | 64.39 ± 19.89 | 65.83 ± 16.79 | 0.633 |
| **RBC-o-α-Syn in patients with different sexes (ng/mg)** | | | |
| Male | 60.47 ± 17.07 | 63.40 ± 15.45 | 0.441 |
| Female | 67.56 ± 21.40 | 68.61 ± 17.80 | 0.823 |
| **RBC-o-α-Syn in patients stratified by age (ng/mg)** | | | |
| < 65 (y) | 64.35 ± 18.19 | 67.14 ± 15.75 | 0.498 |
| ≥ 65 (y) | 64.43 ± 21.55 | 64.76 ± 17.54 | 0.941 |
| **RBC-o-α-Syn in patients stratified by age at onset (ng/mg)** | | | |
| < 60 (y) | 64.61 ± 19.34 | 67.77 ± 15.65 | 0.461 |
| ≥ 60 (y) | 64.18 ± 20.40 | 64.13 ± 17.56 | 0.992 |
| **RBC-o-α-Syn in patients stratified by disease duration (ng/mg)** | | | |
| 0-2 (y) | 64.62 ± 20.14 | 65.61 ± 13.41 | 0.858 |
| 3-5 (y) | 68.96 ± 20.49 | 65.42 ± 17.47 | 0.533 |
| 6-10 (y) | 58.60 ± 17.67 | 67.88 ± 20.81 | 0.126 |
| > 10 (y) | 70.56 ± 15.44 | 63.01 ± 9.38 | 0.397 |
| **RBC-o-α-Syn in patients stratified by H＆Y stages (ng/mg)** | | | |
| 1 | 65.05 ± 17.13 | 60.25 ± 13.93 | 0.453 |
| 2 | 66.46 ± 21.64 | 66.37 ± 17.68 | 0.984 |
| 3 | 57.80 ± 19.21 | 72.13 ± 13.97 | **0.032** |
| 4-5 | 60.92 ± 14.69 | 51.70 ± 10.27 | 0.407 |

*RBD, Rapid eye movement sleep behavior disorder; RBD (-): RBDQ-HK scores < 19; RBD (+): RBDQ-HK scores ≥ 19. Bold, p < 0.05.*

**Supplementary Table 3 |** Overall and clinical variable-stratified comparisons of RBC-o-α-Syn levels in PD patients with or without dysosmia.

| Variables | Dysosmia (-) | Dysosmia (+) | *P-value* |
| --- | --- | --- | --- |
| **Demographic and clinical data** | | | |
| Sex (male/female) | 46/58 | 34/29 | 0.264 |
| Age (y) | 64.1 ± 7.9 | 63.4 ± 9.8 | 0.598 |
| Age at onset (y) | 59.7 ± 8.9 | 58.5 ± 10.2 | 0.434 |
| Disease duration (y) | 4.4 ± 4.0 | 4.9 ± 3.8 | 0.477 |
| H＆Y stage | 2 (1, 2) | 2 (1, 2) | 0.717 |
| **RBC-o-α-Syn in patients (ng/mg)** | | | |
| RBC-o-α-Syn (ng/mg) | 64.76 ± 18.36 | 65.19 ± 19.55 | 0.886 |
| **RBC-o-α-Syn in patients with different sexes (ng/mg)** | | | |
| Male | 62.69 ± 15.57 | 60.30 ± 17.55 | 0.527 |
| Female | 66.40 ± 20.15 | 70.93 ± 20.19 | 0.332 |
| **RBC-o-α-Syn in patients stratified by age (ng/mg)** | | | |
| < 65 (y) | 66.80 ± 16.77 | 62.56 ± 18.29 | 0.299 |
| ≥ 65 (y) | 62.64 ± 19.64 | 67.44 ± 20.29 | 0.285 |
| **RBC-o-α-Syn in patients stratified by age at onset (ng/mg)** | | | |
| < 60 (y) | 67.85 ± 17.89 | 62.26 ± 18.05 | 0.186 |
| ≥ 60 (y) | 61.90± 18.32 | 67.86 ± 20.45 | 0.167 |
| **RBC-o-α-Syn in patients stratified by disease duration (ng/mg)** | | | |
| 0-2 (y) | 65.72 ± 17.96 | 62.72 ± 20.05 | 0.579 |
| 3-5 (y) | 67.48 ± 18.75 | 67.47 ± 20.05 | 0.999 |
| 6-10 (y) | 60.27 ± 19.09 | 64.63 ± 19.64 | 0.469 |
| > 10 (y) | 60.20 ± 14.86 | 69.61 ± 18.26 | 0.276 |
| **RBC-o-α-Syn in patients stratified by H＆Y stages (ng/mg)** | | | |
| 1 | 66.89 ± 17.07 | 59.23 ± 14.62 | 0.151 |
| 2 | 65.46 ± 19.66 | 67.94 ± 20.70 | 0.585 |
| 3 | 62.72 ± 16.50 | 68.06 ± 22.13 | 0.482 |
| 4-5 | 48.46 ± 3.81 | 61.01 ± 14.94 | 0.263 |

*Dysosmia (-): AHRS scores ≥ 22; Dysosmia (+): AHRS scores < 22.*

**Supplementary Table 4 |** Overall and clinical variable-stratified comparisons of RBC-o-α-Syn levels in PD patients with or without constipation.

| Variables | Constipation (-) | Constipation (+) | *P-value* |
| --- | --- | --- | --- |
| **Demographic and clinical data** | | | |
| Sex (male/female) | 41/48 | 39/39 | 0.644 |
| Age (y) | 61.0 ± 8.8 | 67.1 ± 7.2 | **< 0.0001** |
| Age at onset (y) | 57.1 ± 10.1 | 61.7 ± 7.9 | **0.001** |
| Disease duration (y) | 3.9 ± 3.7 | 5.4 ± 4.1 | **0.019** |
| H＆Y stage | 2 (1, 2) | 2 (2, 3) | **0.011** |
| **RBC-o-α-Syn in patients (ng/mg)** | | | |
| RBC-o-α-Syn (ng/mg) | 67.09 ± 19.58 | 62.45 ± 17.57 | 0.113 |
| **RBC-o-α-Syn in patients with different sexes (ng/mg)** | | | |
| Male | 64.85 ± 18.18 | 58.34 ± 13.71 | 0.079 |
| Female | 69.01 ± 20.52 | 66.55 ± 19.89 | 0.579 |
| **RBC-o-α-Syn in patients stratified by age (ng/mg)** | | | |
| < 65 (y) | 65.10 ± 17.05 | 65.73 ± 18.26 | 0.880 |
| ≥ 65 (y) | 70.48 ± 22.87 | 60.80 ± 16.98 | **0.030** |
| **RBC-o-α-Syn in patients stratified by age at onset (ng/mg)** | | | |
| < 60 (y) | 65.63 ± 16.99 | 65.97 ± 20.04 | 0.938 |
| ≥ 60 (y) | 69.05 ± 22.45 | 60.36 ± 15.56 | **0.038** |
| **RBC-o-α-Syn in patients stratified by disease duration (ng/mg)** | | | |
| 0-2 (y) | 66.00 ± 20.78 | 63.16 ± 14.49 | 0.568 |
| 3-5 (y) | 70.33 ± 20.95 | 63.18 ± 15.74 | 0.209 |
| 6-10 (y) | 63.91 ± 14.33 | 60.91 ± 22.13 | 0.621 |
| > 10 (y) | 67.24 ± 13.70 | 63.83 ± 10.44 | 0.685 |
| **RBC-o-α-Syn in patients stratified by H＆Y stages (ng/mg)** | | | |
| 1 | 66.50 ± 17.21 | 57.70 ± 13.02 | 0.125 |
| 2 | 68.73 ± 21.96 | 64.37 ± 18.06 | 0.324 |
| 3 | 66.34 ± 17.57 | 62.06 ± 19.15 | 0.536 |
| 4-5 | 49.69 ± 0.13 | 58.51 ± 14.93 | 0.496 |

*Constipation (-): less than one term on the Diagnostic Criteria (ROME III); Constipation (+): more than two terms on the Diagnostic Criteria (ROME III). Bold, p < 0.05.*

**Supplementary Table 5 |** Overall and clinical variable-stratified comparisons of RBC-o-α-Syn levels in PD patients with or without depression.

| Variables | Depression (-) | Depression (+) | *P-value* |
| --- | --- | --- | --- |
| **Demographic and clinical data** | | | |
| Sex (male/female) | 54/58 | 26/29 | > 0.999 |
| Age (y) | 63.5 ± 9.2 | 64.5 ± 7.4 | 0.498 |
| Age at onset (y) | 58.9 ± 9.7 | 59.9 ± 8.8 | 0.527 |
| Disease duration (y) | 4.6 ± 3.8 | 4.6 ± 4.3 | 0.980 |
| H＆Y stage | 2 (1, 2) | 2 (2, 3) | 0.076 |
| **RBC-o-α-Syn in patients (ng/mg)** | | | |
| RBC-o-α-Syn (ng/mg) | 65.24 ± 18.52 | 64.29 ± 19.39 | 0.761 |
| **RBC-o-α-Syn in patients with different sexes (ng/mg)** | | | |
| Male | 60.84 ± 16.29 | 63.41 ± 16.73 | 0.520 |
| Female | 69.33 ± 19.50 | 65.07 ± 21.46 | 0.361 |
| **RBC-o-α-Syn in patients stratified by age (ng/mg)** | | | |
| < 65 (y) | 67.09 ± 17.15 | 60.96 ± 17.39 | 0.151 |
| ≥ 65 (y) | 63.24 ± 19.69 | 66.86 ± 20.43 | 0.429 |
| **RBC-o-α-Syn in patients stratified by age at onset (ng/mg)** | | | |
| < 60 (y) | 67.78 ± 18.29 | 60.72 ± 16.79 | 0.118 |
| ≥ 60 (y) | 62.60 ± 18.39 | 66.85 ± 20.68 | 0.330 |
| **RBC-o-α-Syn in patients stratified by disease duration (ng/mg)** | | | |
| 0-2 (y) | 65.27 ± 17.37 | 64.03 ± 21.08 | 0.813 |
| 3-5 (y) | 67.71 ± 19.05 | 67.00 ± 19.94 | 0.905 |
| 6-10 (y) | 62.86 ± 20.45 | 60.44 ± 16.90 | 0.707 |
| > 10 (y) | 63.65 ± 8.28 | 66.78 ± 14.87 | 0.700 |
| **RBC-o-α-Syn in patients stratified by H＆Y stages (ng/mg)** | | | |
| 1 | 64.81 ± 17.65 | 61.81 ± 12.92 | 0.616 |
| 2 | 65.45 ± 18.84 | 68.29 ± 22.24 | 0.543 |
| 3 | 65.09 ± 19.65 | 62.55 ± 15.57 | 0.732 |
| 4-5 | 66.86 ± 12.15 | 49.98 ± 9.77 | 0.111 |

*Depression (-): HAMD scores < 8; Depression (+): HAMD scores ≥ 8.*

**Supplementary Table 6 |** Overall and clinical variable-stratified comparisons of RBC-o-α-Syn levels in PD patients with or without anxiety.

| Variables | Anxiety (-) | Anxiety (+) | *P-value* |
| --- | --- | --- | --- |
| **Demographic and clinical data** | | | |
| Sex (male/female) | 36/49 | 44/38 | 0.165 |
| Age (y) | 63.3 ± 9.8 | 64.4 ± 7.2 | 0.391 |
| Age at onset (y) | 58.9 ± 10.6 | 59.6 ± 8.0 | 0.673 |
| Disease duration (y) | 4.3 ± 3.8 | 4.9 ± 4.1 | 0.382 |
| H＆Y stage | 2 (1, 2) | 2 (2, 3) | 0.073 |
| **RBC-o-α-Syn in patients (ng/mg)** | | | |
| RBC-o-α-Syn (ng/mg) | 64.94 ± 17.57 | 64.00 ± 20.02 | 0.988 |
| **RBC-o-α-Syn in patients with different sexes (ng/mg)** | | | |
| Male | 60.88 ± 16.88 | 62.33 ± 16.12 | 0.700 |
| Female | 67.93 ± 17.47 | 67.88 ± 23.40 | 0.991 |
| **RBC-o-α-Syn in patients stratified by age (ng/mg)** | | | |
| < 65 (y) | 64.63 ± 15.62 | 66.08 ± 19.31 | 0.713 |
| ≥ 65 (y) | 65.28 ± 19.44 | 63.89 ± 20.56 | 0.752 |
| **RBC-o-α-Syn in patients stratified by age at onset (ng/mg)** | | | |
| < 60 (y) | 65.44 ± 16.19 | 66.07 ± 19.92 | 0.879 |
| ≥ 60 (y) | 64.50 ± 18.71 | 63.79 ± 20.05 | 0.866 |
| **RBC-o-α-Syn in patients stratified by disease duration (ng/mg)** | | | |
| 0-2 (y) | 64.94 ± 16.84 | 64.81 ± 20.77 | 0.980 |
| 3-5 (y) | 66.97 ± 19.09 | 67.94 ± 19.59 | 0.863 |
| 6-10 (y) | 62.41 ± 17.53 | 61.85 ± 20.98 | 0.926 |
| > 10 (y) | 65.96 ± 13.30 | 64.33 ± 10.43 | 0.842 |
| **RBC-o-α-Syn in patients stratified by H＆Y stages (ng/mg)** | | | |
| 1 | 67.02 ± 15.87 | 59.49 ± 16.71 | 0.154 |
| 2 | 63.23 ± 18.57 | 69.68 ± 21.07 | 0.142 |
| 3 | 66.03 ± 17.88 | 63.00 ± 18.79 | 0.665 |
| 4-5 | 66.86 ± 12.15 | 49.98 ± 9.77 | 0.111 |

*Anxiety (-): HAMA scores < 7; Anxiety (+): HAMA scores ≥ 7.*
